# Climate Justice in Rural Southeastern United States: A Review of Climate Change Impacts and Effects on Human Health

**DOI:** 10.3390/ijerph13020189

**Published:** 2016-02-03

**Authors:** Kristie S. Gutierrez, Catherine E. LePrevost

**Affiliations:** 1 Department of Science Education, North Carolina State University, Raleigh, NC 27695, USA; 2 Department of Applied Ecology, Center for Human Health and the Environment, North Carolina State University, Raleigh, NC 27695, USA; celeprev@ncsu.edu

**Keywords:** climate change, climate justice, human health, rurality, vulnerable populations

## Abstract

Climate justice is a local, national, and global movement to protect at-risk populations who are disproportionately affected by climate change. The social context for this review is the Southeastern region of the United States, which is particularly susceptible to climate change because of the geography of the area and the vulnerabilities of the inhabiting populations. Negative human health effects on variable and vulnerable populations within the Southeast region due to changing climate are concerning, as health threats are not expected to produce parallel effects among all individuals. Vulnerable communities, such as communities of color, indigenous people, the geographically isolated, and those who are socioeconomically disadvantaged and already experiencing poor environmental quality, are least able to respond and adapt to climate change. Focusing on vulnerable populations in the Southeastern United States, this review is a synthesis of the recent (2010 to 2015) literature-base on the health effects connected to climate change. This review also addresses local and regional mitigation and adaptation strategies for citizens and leaders to combat direct and indirect human health effects related to a changing climate.

## 1. Introduction

### 1.1. The Vulnerable Southeastern United States

The National Research Council’s *Committee on Understanding and Monitoring Abrupt Climate Change and its Impacts* acknowledges that climate change is most likely occurring at least as fast as any other warming event over the last 65 million years and that this warming trend is expected to increase in its speed and intensity over the next 30 to 80 years [1]. The *Committee* addresses two global changes that are occurring abruptly: the disappearance of Arctic sea ice and increases in the extinction rates of marine and terrestrial species. It is important to not only consider global impacts of climate change but to also address more regionally-based past, present, and future impacts. 

The Southeast region of the United States (U.S.) is exceptionally vulnerable to climate change-related events, such as sea level rise, heat waves, hurricanes, and drought, due to latitude, topography, and proximity to the Atlantic Ocean and Gulf of Mexico [2]. As a result of the changing climate, populations living along the Atlantic and Gulf coasts will be affected by sea level rise and subsequent land loss, and many inhabitants of the Southeast will experience increasing temperatures and more frequent, intense, and sustained extreme heat events [2]. Further, the region’s water supply stress due to periodic water shortages is anticipated to increase significantly by 2050, affecting forestry, recreation, manufacturing, agriculture, power generation, and fisheries [3]. People living within geographically isolated areas in the Southeast are likely to experience the direct and indirect effects of a changing climate differently, and perhaps more dramatically, than those residing in other areas [4]. 

Beyond the susceptibility of the region to climate change-related events due to geography, Southeastern U.S. populations can be characterized as vulnerable due to their rurality and socioeconomic status. There are varying definitions of what it means to be “rural.” The U.S. Census Bureau identifies a rural area to consist of all “territories, populations, and housing units located outside urbanized areas and urban clusters (*i.e.*, densely populated areas containing more than 50,000 or more than 2500 people, respectively)” [1]. At least one-third of the populations in 7 out of the 10 states comprising the Southeastern U.S. live in an area considered rural by the U.S. Census Bureau [5]. The Southeastern U.S. is comprised of over 90% rural land area [5]. People who live in rural areas in the Southeast currently do and will continue to experience climate change in unique ways by virtue of regional changes in temperature, precipitation, and severe weather events and their vulnerable status [5]. 

The USDA Economic Resource Service reports a difference of over $10,000 in per capita income between urban ($32,007) and rural ($21,005) residents [6]. Nine out of the 10 states with the highest rural and small town poverty rates, as calculated from the 2010 U.S. Census, are located in Southeastern or bordering states [7]. Furthermore, Lal *et al.* notes that the Southeastern U.S. has been “plagued by unemployment” [8] (p. 823). These economic conditions may lower this population’s ability to respond to the impacts of climate change. 

The racial and ethnic make-up of the region’s inhabitants is variable throughout the Southeastern U.S., with overall higher percentages of Caucasians, followed by African Americans and then Latinos/Hispanics. Caucasians comprise anywhere from 53% (Georgia) to 92% (West Virginia) of the population in the Southeastern states. Several Southeastern states have larger populations of traditionally underrepresented racial and ethnic groups. While African Americans comprise only 3% of the population of West Virginia, 37% of the population of Mississippi is African American. Latinos/Hispanics comprise 25% of the Florida population [9]. 

### 1.2. Climate Justice

Climate justice, a subset of environmental justice, is defined by Bulkeley *et al.* as the “mobilization of justice with respect to climate policy” [10] (p. 915). Audet argues that climate justice is “socially constructed through conflicts and negotiations” [11] (p. 371). The term “climate justice” is thought to have been coined by Henry Shue in 1992 in *The International Politics of the Environment* text by Hurrell and Kingsburgy [12]. However, the use of the term did not become popularized until the beginning of the 21st century when various activist groups around the world began voicing the perceived injustice of those suffering the most from climate change. This included populations who were living in low lying areas subject to flooding and sea level rise and people living in drought-stricken regions of the globe [13]. 

Climate justice is generally discussed in the literature in relation to one of two types: distributive (outcome) and procedural (process) [14,15]. Bulkeley *et al.* organized distributive justice as the rights and responsibilities of individuals, organizations, and governments within the larger climate justice scene as differentially distributed across space and time (e.g., who is responsible for reducing greenhouse gas emissions and on what time scale should the mitigation be addressed); whereas procedural justice is the inclusion and participation, equitably, of all affected parties (e.g., explicitly determining who should take responsibility for decision making over mitigation policy, how, and for whom is it being done because all parties involved have rights that need to be respected through acknowledgement and voluntary participation) [10]. Fraser also presented a third type, “recognition justice,” where rights and differences of cultural and social groups are recognized and addressed [16]. In this regard, recognition justice calls for the affirmation of difference—based on race, gender, class, and even geography—to overcome obstacles for any subset of the population to be involved in responding to an issue [16]. This review frames climate justice within the Southeastern U.S. in respect to recognition justice. 

To date, issues of equity and climate justice have been more fully investigated at the country-to-country level than at smaller sub-national levels [17]. Concern regarding this relative inattention to vulnerable populations within the U.S. led to the establishment of Executive Order 12898 by President Bill Clinton in 1994 directing “Federal Actions to Address Environmental Justice in Minority Populations and Low-income Populations” [18]. This action resonates with Ebi, Lindgren, Suk, and Semenza’s assertion that the degree to which health risks affect humans is determined not only by the changing climate but also by who and what is exposed and the population’s vulnerabilities [19]. Vice Admiral Vivek H. Murthy, the Surgeon General of the United States, remarked at the Summit on Climate Change and Health (23 June 2015) “Climate change is therefore not just a health issue but a moral issue and each of us has the responsibility to do what we can, as much as we can, for as long as we can.” 

While issues of climate justice have traditionally focused on urban populations, the National Institute of Environmental Health Sciences (NIEHS) identifies serious concerns for vulnerable populations within rural regions in the U.S. because climate change health threats are not expected to affect all populations equally [20]. Lal *et al.* assert that “the literature specifically related to how climate change will affect rural communities, their resilience, and adaptive capacity in the United States is scarce” [8] (p. 819). Further, the literature suggests that vulnerability differs not only among nations and regions but also across demographic divides (*i.e.*, race, ethnicity, age, income level, and gender) [21,22,23]. Beyond the well-established disparities in health between Caucasians and racial and ethnic minorities, rural minorities are at an even greater disadvantage for health status and health care [24,25,26,27]. NIEHS also identifies people living in poverty and outdoor workers (*i.e.*, farmers and farmworkers) as vulnerable populations for climate change effects [20]. Rural and underrepresented populations, such as those inhabiting the Southeastern U.S., represent some of the most vulnerable of demographic and occupational groups. 

### 1.3. Regional Context and Review Framework

This review consists of three primary sections. The first addresses evidence of existing and projected climate change effects in the Southeastern U.S. The second section links climate change to human health, with a focus on rural Southeastern U.S. communities. The third makes suggestions for climate change mitigation and adaptation in the Southeastern U.S., differentiating strategies at the local, regional, and national level. Throughout the review, there is an emphasis on the Southeastern U.S. because of the vulnerabilities of the region’s populations due to both their rurality and socioeconomic status, as well as the geographic features of the region that are expected to exacerbate climate change-related impacts [2].

## 2. Evidence and Projected Climate Change Effects in the Southeastern United States 

**Figure 1:**
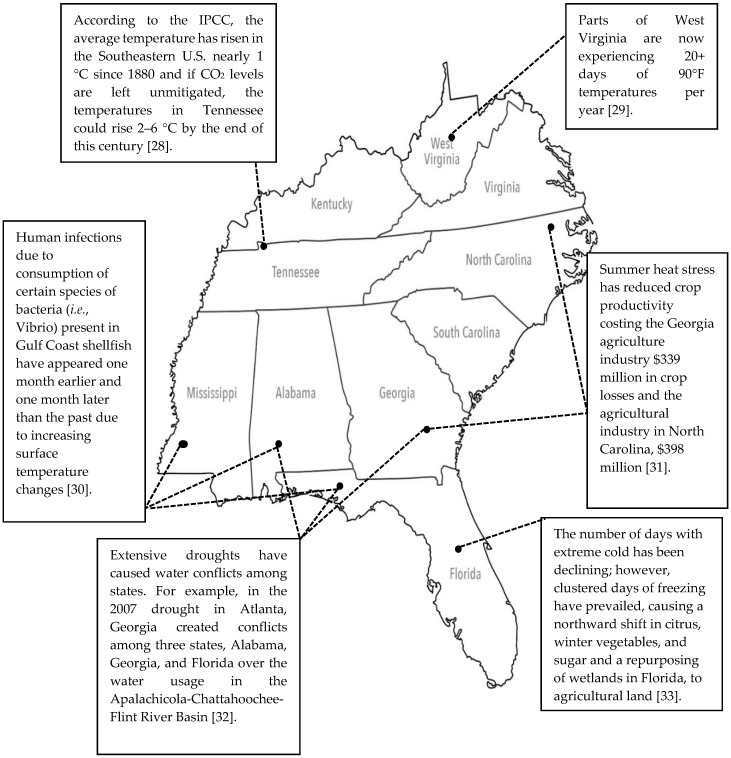
Map of Southeastern U.S. region with corresponding climate change effects identified.

Figure 1 shows the states comprising the Southeastern U.S. and specific effects of climate change in the targeted region. Climate change is already evident through physical climatic data taken over the last half century, and impacts have been documented and are expected to continue, if not worsen. Table 1 outlines four major subsets of climate change: temperature, precipitation, sea level rise, and extreme weather events. The evidence of climate change presented is specific to the Southeastern U.S., and projected climate change effects for both urban and rural Southeastern populations from the literature are identified. Literature often emphasizes the impacts of climate change in relation to large urban centers in developed countries, such as the U.S., and overlooks surrounding populations of rural communities in these same countries. 

**Table 1:** Climate change in in the Southeastern U.S.: Evidence supporting climate change and past and potential population impacts.

Environmental Factors and Supporting Scientific Data	Historical Evidence of Impacts	Predicted Future Impacts
**Temperature**		
***Extremes***		
*Extreme Hot*— Since 1970 there have been increasing numbers of days above 95 °F and nights above 75 °F, and decreasing numbers of extremely cold days [34].	Major cities such as Atlanta, Miami, New Orleans, and Tampa have reported an increase in deaths from 1975 to 2004 relative to an increase in 95 ^o^F and above days [35].	Heat stress can affect dairy and livestock production and reduce crop productivity with a coupling of drought conditions [36]. Climate projections indicate that Georgia’s corn yields could decline by 15% and wheat yields by 20% by the end of 2020 [37]. Increased temperatures will cause a decline in dissolved oxygen in streams, lakes, and rivers, causing fish kills and loss of aquatic biodiversity [38].
*Extreme Cold*— The Intergovernmental Panel on Climate Change (IPCC) indicates that in the last 50 years, daily minimum temperatures have increased faster than daily mean or maximum temperatures [28]. Thus, the frequency of extreme cold temperatures has declined over the last half-century.	From records throughout the U.S. from 1895–2010, the trends show that in the most recent decades there has been an increase in the number of heat waves and a decrease in the number of cold waves [39]. This has direct impact on agricultural products with temperature threshold responses of agricultural pests [40].	While extreme events will increase overall, some extreme events, such as cold, will decrease [41] (p. 495). The energy infrastructure in the Southeast U.S. can produce 32% of the nation's energy and is currently providing nearly 27%. Net energy demand will increase along with energy costs due to more extreme high relative to low temperatures and increased air conditioning usage [42].
***Seasonal Changes***		
*ENSO* ENSO (El Niño Southern Oscillation), periodic changes in ocean surface temperature in the mid-latitude Pacific Ocean, may be correlated to increased precipitation in North America; whereas, La Niña conditions in the tropical Pacific may be correlated to drought conditions in North America. These events are thought to be closely tied to global warming [26].	The biggest cause of flooding and drought worldwide can be attributed to El Niño and La Niña climatic events [43]. The El Niño/La Niña event in 1997–1998 caused $35–45 million in damage and approximately 23,000 deaths worldwide as the Southeastern U.S. received record-setting rainfall [44,45].	Atmospheric modelling programs project an increase of El Niño events due to global warming, increasing occurrences of devastating weather effects [44]. Climate change may have a significant impact on the processes and feedbacks that are responsible for determining ENSO characteristics and thus, the frequency and strength of events [46].
*Changes in Seasons* Records from 1948 to 2007 have shown intensified fluctuations in summer rainfall in the Southeastern U.S. These anomalies are due to an increase in dry days occurring in already dry summers and an increase in wet days occurring in an already wet summers [47]. The increased evapotranspiration occurring in extensive droughts increase sensible heat fluxes and surface temperatures, intensifying summer heat waves [48].	In the Southeast region, mortality rates due to seasonal heat sensitivity have remained consistent over recent time between 0.5 and 1.0 DSM (deaths per standardized million), and national regional variability in mortality has become less apparent across the U.S. [35].	Crops from trees and bushes requiring chilling periods may need to be replaced due to seasonal changes [49]. Warming in the Northern portion of the Southeast U.S. is projected to increase the length of the “freeze-free” season by as much as 30 days by approximately 2050 [31].
**Precipitation**		
***Flooding*** The IPCC shows that incidences of flooding, as well as drought, have increased substantially in the last three decades [28].	In Summer 2011, the Lower Mississippi Valley experienced areas of flooding and drought related to the La Niña Conditions in the Pacific Ocean [31]. In Baton Rouge people were piling sandbags to protect from flooding, while people living in the upper landscape in Louisiana experienced extreme drought [31].	National Oceanic and Atmospheric Administration‘s (NOAA) Climatic Data Center expects the Southeastern U.S. to remain on an increasing participation trend except in summer months [31]. Rural areas may be slightly better suited for flooding conditions due to pervious land cover by forested and agricultural land as compared to an impervious urban environment [50].
***Drought*** In the Southeastern U.S., the percentage of areas experiencing moderate to severe drought conditions has increased in the past 30 years [38].	Drought and intense thunderstorms have contributed to an increase in soil runoff and erosion and have affected crop yields as a result [51].	The Southeast is in a transition zone between conditions projected to be wetter in the north and drier in the southwest [34]. The net water availability in the Southeast U.S. is projected to decrease in the decades to come, this is particularly in the western Southeast [52]. The North Atlantic Subtropical High (NASH) will intensify and move westward with the increasing CO_2_ levels, increasing both extreme rainfall and drought in the Southeastern U.S. [53].
***Storm Surge*** Empirical evidence through modelling shows a heightened Atlantic hurricane surge in warmer conditions that is further exacerbated by sea level rise [54].	The North Carolina Department of Transportation is already raising U.S. Hwy 64 from Albemarle-Pamlico by four feet to allow for anticipated sea level rise water levels and subsequent storm surges [55].	Land loss may cause loss of plant and wildlife, food security, connectivity to the mainland, connections among family members and community cohesiveness [2]. Homes and infrastructures are susceptible to sea level rise, indirectly causing an increase in insurance cost or inability to receive coverage in certain areas such as along the Gulf Coast [56].
***Saltwater Intrusion*** This occurs when an aquifer is pumped faster than can be replaced and saltwater moves into the aquifer. The effects of groundwater extraction due to population growth along coastlines on coastal aquifers is more significant than the impact on sea-level rise due to climate change. Salt water inundation (landward movement of the coastline) will be more important than salt water intrusion due to sea-level-rise [57].	Rising sea levels put additional stress on energy and water utility companies to guard against contamination of saltwater into freshwater reserves along the Atlantic and Gulf coasts [2]. Leaders in Hallandale Beach, Florida, have already closed down 6 of the 8 drinking water wells due to salt water intrusion from seawater moving into porous aquifers [58,59].	Crop production will be reduced due to the availability of freshwater underground for irrigation as saltwater intrudes aquifers in times of drought [60]. The development of lands due to increasing populations will exacerbate saltwater intrusion into freshwater aquifers, rendering the aquifer useless for irrigation and household use [61].
***Sea Level Rise*** Sea level has risen globally an average of 8 inches over the last century [62].	Louisiana has lost 1880 sq mi of land in the last 80 years due to rising seas, sinking lands, and human development [2].	Sea level rise is expected to continue in this trend indefinitely [62]. Municipal infrastructures such as cities, railways, airports, and water supplies are at low elevation and subject to sea level rise. New Orleans, Miami, Tampa, Charleston, and Virginia Beach are particularly susceptible to sea level rise [63]. In the land areas bordering the Gulf of Mexico, almost all of the “most socially vulnerable people live in areas unlikely to be protected from inundation“ due to sea level rise [64].
**Extreme Weather Events**		
**Hurricanes** Florida experienced four major hurricanes in one month in the summer of 2004, and the 2005 hurricane season brought four additional major hurricanes [65]. Between 1994 and 2008, rainfall from U.S. tropical cyclones that made landfall was higher than the historical average [66].	Hurricane-associated winds and flooding not only damaged property but caused drowning, injury, stress, illness, and death due to contaminated floodwater and CO poisoning (from generator use) [65]. “The Southeast has been affected by more billion-dollar disasters than any other region“ [2] (p. 397).	Projections suggest that warming will cause few tropical storms and hurricanes; however, the storms that do form will increase in intensity (*i.e.*, more Category 4 and 5 Storms). There may be even greater economic repercussions for those living within the paths of hurricanes [67].
**Tornadoes** There has been an increase in number of tornadoes over the last 50 years but the increase is not statistically significant [34].	The Southern U.S. experienced 753 tornadoes in April, 2011, breaking the previous monthly record of 542. In May 2011, 178 fatalities were reported in relation to tornadoes in the Southern U.S. [68].	Conditions leading to strong thunderstorms and subsequently, tornadoes, are expected to increase with warming; however, there are other factors to consider such as vertical and horizontal wind variations that are needed to produce tornadoes [43].
**Winter Storms** The number of severe snowstorms since 1960 is more than twice that of severe regional storms that occurred in the 60 years prior [34].	Direct relationships between human health and cold temperature are not as pronounced as compared to hot temperatures; thus, linking cold weather and death rates has been more difficult [69].	Climate change will not only alter globally averaged surface temperature but also changes atmospheric circulation; occasionally stronger winds from polar regions will cause colder winters in the Southeastern U.S. [43]. Power outages associated with winter storms may lead to an increase in air quality problems from CO and particulates from wood and coal-burning stoves and fireplaces as well as gas or diesel generators [70].
**Thunderstorms** Severe thunderstorms with large values of wind shear and potential energy and moist enthalpy close to the Earth‘s surface have been increasing over the last few decades [34].	Wildfires are often begun by lightning strikes; the Southeast U.S. has the highest frequency of lightning strikes in the nation [71].	Due to increasing temperatures and a change in weather patterns, lightning frequency may increase, which will, in turn, affect air quality and increase the occurrences of direct lightning strikes and wildfires [2].

From Table 1, it is clear that climate change is underway in the Southeastern region of the U.S. and that this change has had and will continue to have a direct impact on human populations. The projected increase in hurricane intensity will undoubtedly be one of the most challenging climate change effects for vulnerable populations throughout this region as many already-vulnerable populations live within low-lying areas along the coast. One has only to recall the devastation of Hurricane Katrina, which hit the Gulf Coast Southeastern states in 2005, to understand the potential loss of built capital (*i.e*., residential, commercial, agricultural and industrial infrastructure), human capital (*i.e*., life, physical and mental health), and social capital (*i.e*., social supports and networks, emergency funds) [72]. 

Temperature extremes and seasonal shifts will also present wide-ranging challenges to the vulnerable populations inhabiting the Southeastern U.S. Extreme hot temperatures will not only directly affect human health through heat exhaustion and heat stroke, particularly among manual laborers [73], but will also indirectly affect health through reduced yields for agricultural crops and loss of aquatic life. While much of the existing literature on climate change effects focuses mainly on large populations in urban centers and impoverished people in less economically developed countries, the literature highlighted in Table 1 points to future concerns, particularly for rural and vulnerable populations in more economically developed regions, such as in the Southeastern U.S., in the areas of human health and well-being.

## 3. Climate Justice and Human Health

Issues of climate justice have been explored at the global level, comparing impacts on developed countries (DCs) (e.g., U.S., Japan, Germany, France, United Kingdom) and least developed countries (LDCs) (e.g., Ethiopia, Rwanda, Yemen, Haiti) [74,75,76]. However, the vast discrepancies in economic conditions and health status and, as a result, environmental justice and equity within a single DC or LDC country are not as well understood. Marmot, Allen, Bell, Bloomer, and Goldblatt emphasize that *all countries* should have two aims in regard to human health: (1) improve the average health of its citizens and (2) reduce health inequities among low-, middle-, and high social classes to the health level of the most advantaged [77]. This makes it particularly concerning that as a result of climate change impacts the inequality of health status and healthcare access by socioeconomic class is expected to worsen [78]. 

**Figure 2:**
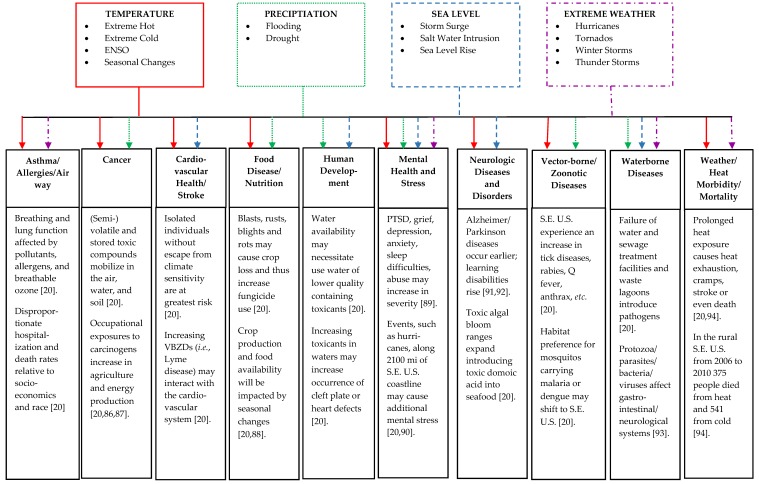
Health effects of climate change in vulnerable populations in the Southeastern U.S. connected to temperature, precipitation, sea level change, and extreme weather events.

People inhabiting rural areas within the U.S. have reduced longevity as compared to their urban counterparts; Singh and Siahpush determined that life expectancy was inversely related to rurality and that the disparity between urban and rural communities widened over time [79]. Comparing two extreme life expectancies in the U.S. in regards to rurality, race, and gender, poor African American men in non-metropolitan areas averaged a 67.7 year life expectancy, which is more than 20 years less than equally poor Asian/Pacific Islander women in metropolitan areas (89.6 years) [79]. As suggested by this comparison of life expectancy as an indicator of health, rural and vulnerable populations in the Southeastern U.S. will have unique challenges related to indirect and direct effects of climate change on human health. Figure 2 identifies human health effects related to climate change in rural areas that may further contribute to urban-rural, racial, and income discrepancies.

The range of health impacts identified in Figure 2 highlights the need for continued and expanded climate justice interventions in the Southeastern U.S., where the most vulnerable groups are among the most negatively impacted by the changing climate. Many of the health issues addressed in Figure 2 are related to chemical or biological contaminant exposure as an indirect result of climate change effects, including increasing temperatures and water quality and quantity concerns. For example, increased temperatures associated with climate change are expected to release volatile compounds that were previously trapped in water, such as trichloroethylene (TCE) and polychlorinated biphenyl (PCB), exposing populations through ingestion or inhalation to these and possibly other carcinogenic toxicants [20]. Additionally, flooding may overburden water treatment facilities and waste lagoons from animal agriculture, exposing populations to pathogenic viruses and microorganisms. Flooding will also cause increased pesticide run-off and may introduce fertilizers containing nitrogen and phosphorus into the water, creating harmful non-toxic and toxic algal blooms that may sicken or kill animals and people [20,80].

Figure 2 provides evidence to support the link between environmental factors associated with climate change and effects on human health. McMichael argued that, in addition to large-scale and systemic environmental factors (such as those associated with climate change), demographic and social factors *(i.e*., population growth, aging, family structures) and economic activity (*i.e*., wealth creation and distribution and financial status and health care) impact human population health [81]. While environmental factors—for example, increased foodborne illnesses and waterborne diseases and the depletion of drinking water resources—are the most obvious linkage between climate change and human health, demographic and economic factors also come into play. Human health quality and access to care are directly related to socioeconomic and geographical status [81]. 

Access to services, facilities, and goods has been an overarching theme within the social and environmental justice movements and extends to the realm of climate justice as well, with particular relevance for the Southeastern U.S. Accessibility to health facilities and services often encompasses three categories of access: financial access, behavioral access, and spatial access [82]. *Financial access* is tied to the ability of people to receive adequate health care under personal cost constraints. *Behavioral access* refers to how people utilize health services, including doctor visits, medications, and emergency care. *Spatial access* addresses the geographic distribution and subsequent travel times to health care facilities, especially in remote, rural areas. Health disparities among the most vulnerable rural inhabitants—including children, adolescents, and the elderly—are often attributed to inequity in the distribution of health care providers, especially access to physicians relative to other providers (e.g., nurse practitioners and midwives) [83]. There are areas in the Southeastern U.S., predominantly in rural locations, that are identified as primary care health professional shortage areas (PC-HPSAs) [84]. For example, Thomas *et al.* identified a rural area in Georgia where no pediatricians were accepting new Medicaid (health care program for those with low incomes and limited resources) patients and there was less than one family/general practitioner per every 2000 individuals per county. As the climate-induced health outcomes outlined in Table 2 (e.g., asthma attacks, heat strokes, and psychological stress disorders) increase, the need for health care services that are equitable, affordable, and positioned appropriately for Southeastern rural communities will be essential.

Access to health care facilities and services is not the only environmental justice issue at play; there are additional disparities in access to quality, healthy food sources, which often vary by rurality, ethnicity, and socioeconomics. Access to healthy food options can help populations maintain health in a preventative fashion [85], and Hilmers, Hilmers, and Dave identified potential public health hazards for “low-income and racial/ethnic minority populations” with regard to their ability to make health dietary decisions and to manage a healthy body weight due to a lack of access [85] (p. 1652). As food production shifts locally, nationally, and globally due to temperature and precipitation changes, costs and accessibility of quality foods for all communities may be stressed even further. This stress will likely put an additional burden on low-income, minority, and remote communities to acquire healthy and affordable nutritional options. 

## 4. Mitigation and Adaptation Strategies for the Southeastern U.S.

*Mitigation* aims to reduce human influence on the climate system, whereas *adaptation* encompasses policies and programs that work to prevent or minimize impacts of a changing climate on human populations [95]. Adaptation can be either anticipatory (occurring prior to experiencing effects of climate change) or responsive (resultant from climate change effects already occurring) [96]. Local climate change mitigation efforts may include fundraising for and installation of solar panel farms in support of low-emission, clean-energy power sources. An example of a local adaptive measure to reduce flooding related to climate change is the creation of a sea wall along a beach-front property. Where mitigation approaches naturally take place at all levels *(i.e*., local, regional, national, international), oftentimes adaptation to climate change occurs at the local or regional level [97]. It should be noted, however, that mitigation and adaptation are not mutually exclusive as the two are inherently linked [97].

The U.S. Geological Survey identifies more than 15 federal government entities that have been established to not only research the science behind climate change (e.g., National Oceanic and Atmospheric Association: Climate Division) but also to ensure the well-being of citizens in the wake of climate change (e.g., Centers for Disease Control and Prevention: Climate and Health Division) [98]. However, there is little evidence of positive climate change interventions by the organizations listed, as well as others, especially in rural areas and among underrepresented populations. Bierbaum *et al.* categorizes four groups of participants in climate change mitigation efforts: “federal government, states, tribal and local/regional governments, and private sector and non-governmental organizations” [99] (p. 364). It is recognized that having stakeholders within the private sector who are members of, represent, and/or engage with vulnerable populations take a more active stance and give voice to local citizens and communities serves to guide and benefit the mitigation and adaptation efforts of both public and private entities [99]. For example, the Coastal Sustainability Studio at Louisiana State is helping to implement community-developed plans and training tools for areas impacted by Hurricanes Gustav and Ike, and the Fish and Wildlife Service and The Nature Conservancy have marketing efforts that highlight how various types of stakeholders can positively impact erosion and salt water intrusion. 

The *Panel on Adapting to the Impacts of Climate Change* acknowledges that “there is currently no a clear federal policy directive to encourage proactive adaptation to climate change” and that more needs to be done at the national level “to fill this void” [100] (p. 4). A few suggestions made by the *Panel* include increasing the federal government’s role as a catalyst to provide information, resources, and incentives; evaluate needs for risk management; and serve as a role model for programs nationwide [100]. One of the limiting factors for the availability of solid information about the benefits, costs, potentials, and limits of climate change adaptation actions is the “diversity of impacts and vulnerabilities across the United States” [100]. Greater focus on regional needs and additional involvement of stakeholders impacted by our changing climate would inform mitigation and adaption strategies in the Southeastern region and throughout the U.S.

Globally, there are many mitigation and adaptation policy dilemmas [15]: Who is responsible for climate change impacts? How should DCs assist LDCs adapt to climate change? How should assistance be distributed among LDCs? What are fair procedures for planning and decision making? These questions have relevance intranationally as well. Vulnerable populations in the Southeastern U.S. who are already experiencing the burdens of climate change in the form of climate variability and extreme weather events will require protection and assistance through national policies falling under the umbrella of climate justice. This does not suggest that mitigation and adaption policy response remains exclusively at the national level because, in the case of many climate change adaptations, responses at multiple levels are necessary for change to occur. Table 2 identifies local and regional mitigation and adaptation strategies to address climate change in the Southeastern U.S. with respect to the health effects identified in Figure 2.

**Table 2:** Example climate change mitigation and adaptation strategies addressing health effects by exposure class in the Southeastern U.S.

Exposure Class	Mitigation Strategy Example	Adaptation Strategy Example	Related Health Effects
**Air **	**Ozone:** Local and regional government, as well as private landowners, increase plant and forest coverage to reduce ambient concentrations of ozone. Those in the public and private sector reduce vehicle miles traveled, use alternate fuel types, and carpool in rural and nearby urban areas to minimize release of ozone precursors [20]. **Greenhouse Gases:** Communication technologies such as the Internet, online meeting and conferencing, and document sharing decrease vehicle and air transportation, thus decreasing greenhouse gas emissions and decreasing carbon footprints [101]. Reduced meat consumption may mitigate greenhouse gas emissions but may negatively impact zinc and iron intake, requiring that winter fruit and vegetable supply availability be monitored [102]. Biofuels used as a renewable energy source but must be monitored as they can exacerbate greenhouse gas emissions through their combustion [20]. The IPCC Working Group 2, Fifth Assessment Report, recommends providing better access to reproductive health services to improve both maternal and child health while reducing population growth and subsequently greenhouse gas emissions over time [103]. **Particulate Matter:** Decreases in emissions from transportation sectors result in decreased toxicants such as sulfur oxide and particulate matter (PM), reducing incidences in lung cancer [20]. Changes in agricultural practices such as frequent tillage of land minimizes introduction of airborne particulates, some of which can cause infectious disease [104].	**Communication Methods:** Susceptible and vulnerable populations for breathing-related conditions made aware of weather conditions (*i.e*., extreme temperatures or extreme humidity levels) and adjust activities and locations accordingly [20]. Aggressive public health plans (*i.e*., early warning systems and improved health communications) may prove successful in minimizing heat related mortalities along with maximizing air-conditioning use and sun-shielding/cooling clothing, and decreasing time spent outdoors [20]. **Diet/Behavior Modification:** The reduction of red meat consumption to a more plant protein diet may also lower the risk of colorectal and other cancers, as well as lower the risk of diabetes, obesity, and heart disease [105]. This would provide a co-benefit for individual health, health care costs and mitigate greenhouse gas emissions [106]. Increasing exercise routines and ability to maintain cardiovascular health reduces the burden of cardiovascular disease, especially in the Southeastern U.S.; however, individuals need access to indoor exercise equipment as outdoor air pollution levels are expected to rise. Populations already financially stressed are least likely to have consistent access to indoor facilities [20]. **Equipment Provisioning:** Heat-related illnesses can be prevented through the access and use of air conditioning and fluid intake in high risk populations [91]. However, the increase in emissions (depending on the power source used) and energy costs associated with increased air-conditioning use requires monitoring [20].	Asthma, Respiratory Allergies, and Airway Diseases. Cancer. Cardiovascular Disease and Stroke. Foodborne Disease and Nutrition. Human Developmental Effects. Mental Health and Stress-Related Disorders. Weather and Heat-Related Morbidity and Mortality.
**Water and Soil**	**Toxicant Exposure:** Reduction in fossil fuel use minimizes the release of some neurotoxicants including arsenic, mercury, and other heavy metals [20]. Proper disposal of products such as florescent light bulbs and batteries for electric vehicles is essential to reduce risk of contamination [20]. Proper agricultural practices such as minimal to no-tillage of land and proper pesticide application reduce erosion and minimize pesticide infiltration into waters and soils [104,107]. By removing toxic compounds from building materials and facilitating recycling and biodegradation of components, human illness through air and waterborne pollution is reduced [104]. Diverting staple crops for use as biofuels may necessitate the use of additional chemicals for crop production [20]. **Disease Transmission:** Reforestation can help with flooding and air quality but be a breeding ground for VBZD and reduce potential agricultural land [20]. Continued development of solar and wind farms minimizes water usage in power production and, in turn, minimizes threats of waterborne disease [20].	**Communication Methods:** It is important to continue developing early warning systems, evacuation plans, and emergency plans and warning systems [108,109]. Planners will need to continue efforts to fortify natural barriers for flooding and erosion, such as wetlands and tidal marches [20]. **Combatting Drought:** Ponds and dams are used widely to manage water supplies, possibly allowing for the spread of certain VBZD [110]. Capture and storage of rainwater may prove excellent breeding grounds for mosquitos harboring VBZD. In addition, pesticide use to control organisms harboring VBZD may introduce potential toxicants into environmental sectors [20]. **Land Use:** Zoning permits are limited for at-risk land [108,109]. Best land-use practices in agriculture and use of locally recycled water (grey-water) slow rates of water table depletion and reduce the impacts of heavy precipitation events that are anticipated in the Southeast [20].	Asthma, Respiratory Allergies, and Airway Diseases. Mental Health and Stress-Related Disorders. Neurological Diseases and Disorders. Vectorborne/Zoonotic Diseases (VBZD). Waterborne Diseases. Weather and Heat-Related Morbidity and Mortality.
**Occupational**	**Commercial Technological Advances:** New technologies in power generation (*i.e*., solar cells, portable electric storage systems for cars/batteries, hydrogen fuel cells) require monitoring for unintended occupational exposure to toxic, cancer-causing materials such as lithium, lead, and cadmium [20]. **Toxicant Exposure:** The proper application and use of pesticides reduce occupational exposure for pesticide applicators despite new and expanded pesticide use [20].	**Communication Methods:** Healthcare providers increase awareness that individuals with chronic conditions such as Parkinson’s disease, Alzheimer’s disease, and epilepsy are predisposed to dehydration, heat exhaustion, and heat stroke [108,111]. **Working Condition Modifications:** Heat exposure and excessive air humidity conditions for outdoor workers and workers without air conditioning addressed by way of frequent breaks that include access to water and air conditioning, shifted work output and hours during the hottest part of the day, and monitoring for workers’ physiological reaction to heat [73].	Cancer. Neurological Diseases and Disorders. Weather and Heat-Related. Morbidity and Mortality.

The interconnectedness of mitigation and adaptation strategies and related health effects is evident from Table 2. Some climate change mitigation and adaptation efforts produce a domino effect, resulting in more positive (e.g., changes in agriculture and livestock production that lead to reduced red meat consumption both positively affect human health and minimize greenhouse gas emissions) and potentially negative (e.g., renewable biofuel production may increase the need for agricultural worker labor, increasing opportunity for excessive heat, humidity, and pesticide exposure) effects on human health. 

Rural populations of the Southeastern U.S. are in a position to participate in target mitigation strategies to reduce the anthropogenic impact of climate change through technological developments, workplace (*i.e*., agricultural and industrial) practices, personal and community behavior changes. However, adaptation strategies may prove challenging due to several factors specific to the Southeastern U.S. and may therefore require assistance from regional and/or national groups. For example, enhanced communication methods were a common theme among adaptation strategies in Table 2. Due to the geographic remoteness of communities and associated lack of communication resources (e.g., telephones, televisions, high-speed Internet, and computers) within many impoverished homes, effective and timely communication of health effects for vulnerable populations is difficult. Similarly, while diet and behavior modifications may be able to provide individuals with healthier lifestyles at the same time as adapting to climate change, these modifications require financial resources that may need to be supplemented by government programs. Lack of adequate transportation affects access to both health care and nutritious foods. State and local governments have been successful in collaborating to improve food access in low-income neighborhoods by introducing mobile markets to bring fresh produce into local communities and incentivizing grocery stores with healthy, low-cost fresh food options to move into low-income communities [85].

While both mitigation and adaptation can be planned at various levels *(i.e*., individual, local, regional, national), successful implementation will require multi-level support. Hallegatte *et al.* contend that climate change policies are best implemented at the local scale by mitigating transportation, energy, water, and building infrastructures to reduce human impacts on greenhouse gas emission and by adapting locally to climate change effects [112]. At the local level, effective solutions will necessitate cooperation among “government, business sectors, individuals, and the collective citizenry acting together” [50] (p. 401). In their review, the authors identified criteria from seminal literature that are necessary for developing local climate change solutions: make the process engaging, present content in an understandable fashion, make the information salient to the stakeholders’ and decision-makers’ lives, and foster affective responses that are both relevant and motivating at an individual level [50].

Involving all stakeholders in mitigation and adaptation efforts can create sustainable community development [97]. It is common for mitigation strategies to be presented as a win-win scenario, often referred to as co-beneficial, in order to attract the interest of all organizations and individuals affected by the policy [113]. For example, through building a solar farm in a community, energy service costs could be minimized for the individual, and local companies who create and then install the solar panels would benefit economically as well. In addition to initiating preventative measures to combat current and future climate change, “a comprehensive approach that considers all stakeholders within different levels, and the availability of resources” is recommended for communities and individuals [97] (p. 290). The authors indicate that the best adaption and mitigation strategies for addressing climate change in low- and middle-income communities must focus not only on specific environmental issues but also social and economic issues. Ebi *et al.* suggest that the capacity to adapt to climate change includes population awareness of the issues; option availability and availability of knowledge, skills, and technology; political drive; human and financial investment capital; and institutional capacity [19]. The literature underscores that continual efforts must be made to provide particularly vulnerable populations with the knowledge, skills, and capital to empower individuals and communities to develop and support local and regional co-beneficial mitigation and adaptation strategies.

## 5. Future Research and Limitations 

Ongoing research is needed in the area of climate justice for rural populations in the Southeastern U.S. and various underrepresented groups in regions throughout the U.S., other DCs, and LDCs. Specifically, issues of procedural climate justice as it relates to voice need to be more fully addressed; as Burnham *et al.* insist, more attention needs to be given to “who sits at the table, how they are allowed to participate, whose knowledge counts, and who gets to define the problem” because to this point, the federal and state entities have been the loudest voices [114] (p. 245). Although this review focused on the Southeastern U.S., it was evident through the literature searches conducted that there were scant articles focused on climate change concerns and adaptive strategies targeting regional areas within larger national contexts. Researchers need to assess and develop climate change mitigation and adaption strategies, especially at the local and individual level, tailored to marginalized and vulnerable communities [115]. As globalization creates an interconnectedness with populations afar, issues of environmental justice related to climate change must continually be monitored among the poor and underrepresented in all countries, in addition to the inhabitants of less developed countries [116].

There are several limitations of this review. First, although the Southeast region of the U.S. is relatively small geographically, the scope of this review is still grand in scale. Thus, the information presented in this article is not exhaustive for every topic presented, but is a synthesis of the literature for each subtopic (*i.e*., climate justice, health effects of climate change, mitigation, adaptation) from extensive Google Scholar searches focused on recent research efforts. Articles within the last five years (2010–2015) were targeted. However, often these articles contained references to seminal work and valuable resources dating back to the 1990s; these sources were also included. All journal articles, both national and international, were considered if they were written in or translated into English. Common search terms used included: climate change in the Southeastern United States, health effects of climate change, rural populations and climate change, vulnerable populations and climate change, as well as combinations of the terms. Articles were filtered by reviewing titles and/or abstracts for relevance. Articles were included if they were specific to the Southeastern U.S. and were published within the target time period. Additionally, grey literature was searched and included, as needed, to provide pertinent current data produced by government entities (e.g., U.S. Census Bureau, U.S. Environmental Protection Agency, U.S. Department of Agriculture). Secondly, as aforementioned, the literature directly targeting climate change effects and projections, as well as climate justice, in the Southeastern U.S. is scant, necessitating extrapolation from studies of urban populations or vulnerable populations in other regions. Finally, although the information contained in this review is crucially important to the human health of the vulnerable and underrepresented populations in the Southeastern U.S., neither the sources nor this article itself is directed toward the audience truly at the heart of the matter—the citizens of the Southeastern U.S. Thus, this research should ultimately be used to further advance the understanding and capacity of individuals within the targeted populations and similar populations worldwide to help develop manageable adaptive strategies to combat the changing local, regional, and global climates.

## 6. Conclusions

By virtue of the fact that they are inhabitants of a developed country, populations in the Southeastern U.S. are at a relative advantage with regard to climate change impacts; globally, vulnerable populations are responding to drought, famine, vector/zoonotic-related disease outbreak, and, more recently, cultural genocide as a result of necessary population relocation [117]. Even so, vulnerable communities of the region struggle to attain environmental justice, specifically the right to a healthy environment. The issue of climate justice is particularly salient to the Southeastern U.S. where the geography of the area and the rurality and socioeconomic status of the population make the region susceptible. As there is no “one size fits all” proposition for addressing the needs and protecting the health of vulnerable communities related to the effects of climate change [117] (p. 209), issues of climate justice for the Southeast U.S. and for specific populations in other regions should be considered and given an active voice. Climate justice cannot be a topic only addressed at the global level. Every population throughout the world will experience effects related to climate change somewhat differently, and it is the responsibility of scientists, policy-makers, businesses, and local communities and individuals to take an active participatory role in climate equity issues.
